# Privacy of single-cell gene expression data

**DOI:** 10.1016/j.patter.2024.101096

**Published:** 2024-11-08

**Authors:** Hyunghoon Cho

**Affiliations:** 1 Department of Biomedical Informatics & Data Science, Yale School of Medicine, New Haven CT 06510, USA; 2 Department of Computer Science, Yale University, New Haven, CT 06520, USA

## Abstract

The possibility that single-cell gene expression datasets could leak information about individuals’ genotypes has been largely unexplored. Walker et al. showed that even noisy genotype predictions derived from these data can be linked to the corresponding genotype profiles with significant accuracy.

## Main text

Sharing genomic data requires careful privacy considerations to minimize potential risks to individuals. The primary concern stems from the fact that genetic sequence is unique to each individual and thus can act as a potent biometric identifier. This uniqueness can enable linkage of samples from the same individual across datasets, increasing the likelihood of re-identification and disclosure of sensitive health information.

The sensitivity of genotypic information is well-established, with as few as 30 to 80 independent single nucleotide polymorphisms (SNPs) being statistically sufficient to uniquely identify an individual.^1^ Rare genetic variants offer even stronger identifying signals. Furthermore, searching for relatives in genealogical databases may reveal real identities behind genotypes, a method that has been successfully used in criminal investigations.^2^ Due to these risks, genomic data sharing is often tightly restricted and governed by stringent access controls to protect individual privacy.

The privacy risks associated with other types of genomic data, such as transcriptomic, proteomic, and metagenomic profiles, are less well understood. The higher levels of inherent noise in these datasets—both biological and experimental—are expected to reduce the extent to which personally identifying information could be reliably extracted from them, if at all. This perception of lower risk has often led to open sharing of these data in public data repositories such as ArrayExpress and Gene Expression Omnibus.

However, a growing body of research indicates that sharing various types of molecular data, even those that do not directly disclose genotypes, may pose greater risks than previously recognized. For instance, quantitative trait loci (QTLs) can be used to infer genotypes from gene or protein expression levels.^3^^,^^4^^,^^5^^,^^6^ Other studies have shown that identifying signals could be extracted from genomic data without directly examining the genotypes, e.g., for microRNA^7^ and metagenomic^8^ data. As novel experimental technologies continue to expand the range of genomic data collected from individuals, it is crucial to enhance our understanding of privacy risks to guide responsible data sharing.

In the November 14 issue of Cell (vol. 187/23) of *Cell*, Walker et al.^9^ present a comprehensive study of privacy risks associated with single-cell transcriptomic data, which characterize gene expression levels at single-cell resolution. This investigation is particularly timely given the rapidly growing use of single-cell RNA sequencing (scRNA-seq) across diverse areas of biology and medicine. The authors report that, despite the higher noise levels inherent in single-cell experiments compared to bulk sequencing, genotypic information can still be extracted from processed gene expression count matrices alone, enabling data linkage across datasets.

The linking strategy studied by Walker et al. begins by constructing a "pseudo-bulk" (i.e., aggregate) gene expression profile for each cell type in an individual’s scRNA-seq sample. They then use various forms of eQTL associations to predict genotypes for each sample to compare with candidate genotype profiles to assess linking accuracy. A match is considered successful if the correct sample is assigned the highest match score among the candidates in at least one cell type. The authors evaluated three approaches for genotype prediction: (1) using significant eQTLs from a matching tissue (e.g., from the GTEx consortium), regardless of cell type; (2) using cell type-specific eQTLs from the corresponding cell type; and (3) using eQTLs that are identified *de novo* in a limited training set, based on a relaxed definition that captures deviations in alternative allele frequencies (AAFs). Across various evaluation settings using the OneK1K^10^ and Lupus^11^ scRNA-seq datasets, which include blood samples from 979 and 258 individuals with 14 annotated immune cell types, respectively, the authors reported consistently high linking accuracy (e.g., over 80% using publicly available eQTLs from GTEx and over 70% using only a single cell type, CD4_NC_). Factors such as disease status of individuals, data preprocessing strategies, and cross-dataset generalization were shown to have a limited impact on linking accuracy. These findings provide strong empirical evidence that single-cell gene expression data contains enough genotypic information to enable data linkage.

While it may seem intuitive that single-cell data, which captures more biological information than bulk data, could enable data linkage—as previously shown for bulk data^3^^,^^4^^,^^5^—this study is the first to demonstrate this through extensive experiments. It also provides insights into several issues unique to single-cell data. For instance, the findings highlight the effectiveness of cell type-specific linking, which is not feasible with bulk profiles. The study also shows how the number of cells within each cell type influences linking accuracy and illustrates the possibility of linking without known eQTLs through the authors’ AAF-based strategy, designed to work with the limited sample sizes typical of scRNA-seq datasets.

A notable finding is that the accuracy of predicted genotypes—rather than linking accuracy—was relatively low, falling below 62% across all strategies considered. This suggests that accurate reconstruction of genotypes at individual genomic positions is not necessary for data linkage. Instead, the study suggests that matching samples can be identified because, on an aggregate level, there are significantly more correctly predicted genotypes than incorrect ones compared to non-matching samples. Consequently, the predicted genotypes from single-cell data are likely to have limited utility for scientific inquiries.

This study raises several intriguing questions for future investigation. While the authors performed genotype prediction separately for each cell type, it would be interesting to explore whether joint prediction across all cell types could improve accuracy. Similarly, incorporating haplotype-level sequence patterns (e.g., linkage disequilibrium) might result in further information leakage, as demonstrated in a recent study for bulk data.^3^ Examining how noise in single-cell experiments impacts linkage could be valuable for developing effective mitigation strategies aimed at obscuring genotypic information in these data. For instance, it would be useful to determine whether replicate samples display distinct sets of variants accurately predicted from gene expression or share a common set of identifying variants linked to informative genes that are less affected by noise.

The real-world implications of these findings must be interpreted in context, taking into account the specific characteristics of the datasets involved. Key considerations include the sensitivity of any associated metadata (e.g., clinical attributes), the availability of eQTL or other scRNA-seq datasets that adversaries could use for training, and the potential influence of differences in sequencing technologies, analysis pipelines, tissue of origin, or ancestry composition. Regular reassessment of these issues would help ensure that data sharing practices stay aligned with the evolving understanding of privacy risks.

Overall, this study underscores the need for greater caution in sharing gene expression data from the rapidly expanding collection of scRNA-seq studies. Our perceptions of noise levels in high-dimensional molecular datasets and their impact on privacy can be misleading and should not be a reason to overlook privacy concerns.

## Declaration of interests

The author declares no competing interests.
